# Propofol for palliative sedation: A systematic review of clinical outcomes, safety, and ethical implications

**DOI:** 10.1177/02692163261465774

**Published:** 2026-07-31

**Authors:** Luís Almeida-Jardim, Paulo Reis-Pina

**Affiliations:** 1 Palliative Medicine Center, Faculty of Medicine, University of Lisbon, Lisboa, Portugal; 2 Acute Palliative Care Inpatient Unit, Local Health Unit of Santa Maria, Lisboa, Portugal

**Keywords:** deep sedation, ethics, medical, palliative care, patient safety, propofol, survival rate, symptom burden, terminal care

## Abstract

**Background:**

Palliative sedation is used to relieve refractory suffering in terminally ill patients. Propofol, an intravenous anesthetic, may offer advantages due to its rapid onset and short half-life.

**Aim:**

To evaluate the role of propofol in palliative sedation, focusing on symptom control, sedation depth, survival time, safety, and ethical implications.

**Design:**

Systematic review registered in PROSPERO (CRD42025643575; February 6, 2025).

**Data sources:**

Scopus, MEDLINE, and Web of Science were searched for English-language studies (2005–2024) on propofol use in adult terminally ill patients. Studies comparing propofol with other sedatives or non-sedated controls were included; those involving anesthetic or intensive care settings were excluded. Risk of bias and certainty of evidence were assessed. Results were synthesized narratively.

**Results:**

Ten non-randomized studies were included (observational, cross-sectional, cohort, case series, and before-and-after designs), mostly of moderate quality, conducted in Europe (*n* = 7) involving 4072 patients (28,3% received palliative sedation), mainly with cancer. Symptom control was reported in 73%–100% of cases, target sedation depth was achieved in 52%–100%, although outcome definitions and measurement methods varied. Survival ranged from 19 h to 38 days. Propofol was used alone (*n* = 4) or with other sedatives (*n* = 6). Respiratory depression was the main safety concern. Ethical issues included absence of explicit consent and rare reports of life-shortening intent.

**Conclusions:**

Propofol may be considered as a rescue option in selected refractory cases managed by experienced teams; however, given the low to very low certainty of available evidence, findings should be interpreted cautiously and supported by institutional protocols and further prospective research.

**What is already known about the topic?**

- Propofol has been used as a sedative agent in palliative care, but its role remains less studied compared to midazolam.
- Concerns exist regarding its safety profile, ethical implications, and the lack of standardized protocols.

**What this paper adds?**

- Provides an updated systematic review synthesizing evidence on propofol use for palliative sedation in adult terminally ill patients.
- Demonstrates that propofol achieved high rates of symptom control and sedation depth but with variable survival outcomes.
- Identifies recurring safety and ethical concerns, particularly respiratory depression, documentation of informed consent, and potential life-shortening effects.

**Implications for practice, theory, or policy?**

- Highlights the need for clinical guidance regarding propofol use as a second- or third-line rescue therapy in refractory cases.
- Underlines the importance of integrating ethical frameworks, proportional sedation principles, and structured documentation into clinical decision-making.
- Identifies priorities for future research, including prospective studies, standardized reporting of dosing and monitoring practices, and inclusion of patients with non-malignant conditions.

## Introduction

### Rationale

The primary goal of care for patients with incurable diseases is to minimize symptoms and optimize quality of life.^1^ While many treatment options exist, some distressing symptoms—such as pain, agitated delirium, dyspnea, and nausea—remain refractory to standard interventions.^2,3^

Palliative sedation aims to relieve refractory suffering through the monitored, proportional use of medications intended to reduce consciousness in patients with life-limiting disease, as defined in the revised European Association for Palliative Care (EAPC) framework.^4^ This practice carries important ethical and social implications requiring careful consideration by patients, families, and healthcare professionals.^2,4,5^ Common agents used in palliative sedation include midazolam, neuroleptics, and barbiturates.^6,7^ In refractory cases, combinations may be required, and when these fail, the anesthetic agent propofol may represent a valuable alternative in selected cases.^6,7^

Propofol (2,6-diisopropylphenol) is a short-acting intravenous anesthetic with sedative and hypnotic properties.^8^ Widely used in surgical and diagnostic procedures, it also exerts antiemetic, bronchodilator, and antipruritic effects and has been employed in managing refractory status epilepticus.^7^ With a rapid onset (≈30 s) and short duration of action (5–10 min), propofol allows precise titration to targeted sedation levels.^5–8^ However, side effects—especially with bolus or high-dose infusion—include hypotension, bradycardia, and respiratory depression.^5–8^ Because of these risks and its intravenous administration, propofol is usually restricted to settings with continuous medical supervision.^3,6^ Nonetheless, its short half-life allows for rapid recovery upon discontinuation, suggesting potential applicability in palliative care.^6,8^

An early systematic review examined publications from 1962 to 2005 addressing propofol use in palliative care.^6^ More recent reviews have examined palliative sedation more broadly, with propofol discussed only peripherally. These include the work of Patel et al.,^3^ who illustrated ethical considerations through a case-based discussion; Arantzamendi et al.,^2^ who reviewed prospective studies published between 2014 and 2019; and Bodnar,^9^ who provided a narrative review specifically focused on propofol use at the end of life.

Despite these contributions, existing reviews have not systematically synthesized propofol-specific evidence across key clinical domains, including sedation depth, survival, safety, dosing practices, and ethical safeguards across diverse care settings. Consequently, important uncertainties remain regarding optimal clinical use and governance of propofol in palliative sedation.

To date, no systematic review has comprehensively synthesized propofol-specific evidence across clinical, safety, and ethical domains within the context of palliative sedation.

### Objectives

This systematic review aimed to synthesize current evidence on the clinical use, outcomes, and ethical implications of propofol in palliative sedation within palliative care settings. Specifically, we examined how propofol has been used, in which clinical contexts, and with what outcomes have been reported.

The research question was structured using a PICO framework, addressing adults with advanced illness (Population), propofol for palliative sedation (Intervention), compared with other sedatives or no sedation where available (Comparator), and outcomes including symptom control, sedation depth, survival, safety, and ethical considerations (Outcomes). The guiding research question was: *What is the evidence regarding the use of propofol for palliative sedation in adults with advanced illness?*

## Methods

This systematic review followed the recommendations of the “Cochrane Handbook for Systematic Reviews of Interventions,”^10^ and was reported in accordance with the “Preferred Reporting Items for Systematic Reviews and Meta-Analyses” (PRISMA) guidelines.^11^

### Eligibility criteria

#### Participants

Adult patients with terminal or incurable illness, including both cancer and non-cancer conditions. Caregiver perspectives were also included when reported in the context of patient care.

#### Interventions/comparators

Studies reporting the use of propofol for palliative sedation, either alone or in comparison with other commonly used sedatives or with non-sedated patients, with evaluation of at least one clinically relevant outcome such as symptom control, level of consciousness, survival time, safety outcomes, or ethical considerations.

#### Outcomes

Studies reporting at least one predefined outcome relevant to the review objectives, including symptom control, level of consciousness (sedation depth), survival time, safety outcomes (e.g. respiratory depression), or ethical considerations (e.g. consent or intention).

#### Study design

Only primary research studies were eligible, irrespective of methodological approach.

#### Exclusion criteria

Articles not written in English; studies without extractable data relevant to the objectives studies without extractable data relevant to the objectives (e.g. absence of reported information on propofol administration, sedation outcomes, safety events, survival, or ethical aspects); studies involving propofol use in anesthetic or intensive care settings, or in pediatric populations; and studies with full texts inaccessible via institutional subscriptions.

Studies from intensive care units were excluded because sedation in this context is often initiated for technical or therapeutic purposes (e.g. ventilator adaptation) and only later transitioned to palliative sedation, which differs conceptually from the primary palliative care intention of symptom relief at the end of life in palliative settings.

### Information sources

Scopus, MEDLINE, and Web of Science were searched for articles published in English between January 1, 2005, and August 31, 2024 (last search date). Although the protocol registered in PROSPERO anticipated searches until December 2024, the final search was completed earlier to align with the study timeline.

### Search strategy

The search included free-text terms, with the following structure: (“palliative care” OR “hospice care” OR “end-of-life care” OR “terminal care”) AND propofol AND (sedat* OR “terminal sedation” OR “palliative sedation”) NOT (“ICU” OR “intensive care” OR “critical care” OR “trauma care”) NOT (review or “systematic review” or “scoping review” or book or “meta-analysis”). Filters applied included language (English) and publication date (2005–2024). The complete search strategies for each database, including platform-specific adaptations, are provided in Supplemental Table 1.

### Selection process

In the initial screening, both authors independently reviewed article titles and abstracts. A list of potentially relevant articles was then compiled, followed by a full-text analysis conducted independently by both authors. Any disagreements regarding study selection or data extraction were resolved through discussion and consensus, based on predefined eligibility criteria. A third reviewer was not required, as consensus was achieved in all cases. The number of disagreements and inter-reviewer agreement statistics (e.g. Cohen’s kappa) were not formally recorded.

### Data collection process

Two independent reviewers collected data from each report. The original authors were not contacted to obtain or confirm the data. No automation tools were used in the process.

### Data items

Data were sought for the following outcomes: (1) symptom control; (2) consciousness level; (3) survival time; (4) propofol utilization; (5) safety considerations; and (6) ethical implications. These could be studied alone, combined, or associated with other health-related outcomes, as well in monotherapy or in combination with other sedatives. All results that were compatible with each outcome domain in each study were sought (for all measures). Since some of the included studies conducted qualitative interviews, this data was also analyzed and discussed.

Data were also sought for other variables, such as: (1) article’s characteristics (e.g. authors, country of origin, year of publication); (2) study design; (3) study objectives; 4) participating setting and sample/patient characteristics; (5) refractory symptoms; (6) intervention; (7) comparator or control; and (8) main findings.

### Study risk of bias assessment

Risk of bias was evaluated using the ROBINS-I Cochrane tool,^12^ for non-randomized studies (*n* = 9), and the Joanna Briggs Institute Critical Appraisal tool,^13^ for case series (*n* = 1). The *robvis visualization tool for risk of bias assessments in a systematic review* was used to create traffic light plots of the domain-level judgments for each individual result.^14^ Both authors participated, independently, in this process. No automation tools were used.

### Effect measures

For each outcome, we accepted the effect measures reported by the authors of the included studies and used them in both the synthesis and presentation of results.

### Synthesis methods

A meta-analysis was not performed. A meta-regression was not feasible due to the small number of studies and the substantial heterogeneity of participants, interventions, and outcomes. The evidence was therefore synthesized narratively.

Both quantitative and qualitative outcomes were extracted. Qualitative findings were limited to two studies that conducted interviews alongside quantitative assessments; these results were narratively summarized but no separate qualitative synthesis (e.g. thematic analysis) was undertaken.

To ensure clarity, findings were grouped and synthesized according to six outcome categories: symptom control, consciousness level, survival time, propofol utilization, safety considerations, and ethical implications. To visually present study findings, we developed data-charting forms presented in Table 1 (study characteristics) and Table 2 (main results).

**Table 1.** Characteristics of the included studies (n = 10).

Authors, country	Study design	Objectives	Setting, population, sample	Refractory symptoms	Scales and tools	Key observations
Lundström et al., Sweden^29^	Case series	To survey indications, doses and effects of propofol in PC patients.	Setting: The specialized PCU of Stockholms Sjukhem Foundation.	Anxiety or agitation with intractable pain (*n* = 22), intractable nausea and vomiting (*n* = 13).	N/A	Patients evaluated by a nurse and physician at 1 h after treatment, then by a nurse or physician at 2, 6, and 12 h, thereafter twice a day by nurse and daily by physician.
			Population: Inpatients with advanced cancer (with a short-expected survival) receiving propofol between 1995 and 2004.			
			Sample: *n* = 35, females 63%, mean age 55 (range 28–84) years. Main cancers: ovarian (20%), prostate (11%), breast (9%), sarcoma (9%), lung (6%), renal (6%), stomach (6%) and uterus (6%).			
Won et al., Korea^30^	Prospective cohort study	To investigate clinical outcomes of PS in a real clinical setting.	Setting: A single hospice unit affiliated with a tertiary cancer center in Korea.	Delirium (*n* = 54), dyspnea (*n* = 18), pain (*n* = 13).	Sedation levels measured with RASS.	- Midazolam was used as first line medication, propofol as second line.
			Population: All consecutive patients with terminal cancer hospitalized between September 2015 and March 2017.			- Vital signs monitored 15 and 30 min after injection, then, periodically.
			Sample: *n* = 306 (males 68%, mean age 65.8 ± 11.8 years). From these, 89 (29%) received PS: IPS (*n* = 61) and CPS (*n* = 28). Main cancers: lung (26%), pancreatobiliary (19%) and gastric (13%).			
Kuragaichi et al., Japan^31^	Cross-sectional study	To clarify the current status of PC for heart failure patients in Japan.	Setting: All Japanese Circulation Society-authorized cardiology training hospitals between August and December 2016.	Dyspnea (*n* = 482), anxiety (*n* = 372), depression (*n* = 322), malaise (*n* = 298), anorexia (*n* = 259), insomnia (*n* = 254), pain (*n* = 182), edema (*n* = 154), nausea (*n* = 122).	N/A	Dexmedetomidine (*n* = 133) was most often used, then midazolam (*n* = 116), then propofol (*n* = 80).
			Population: patients diagnosed with terminal stage heart failure.			
			Sample: 544 (54%) institutions returned the questionnaire, 403 (76%) prescribed drug therapy as part of PC.			
Fredheim et al., Norway^32^	Retrospective observational study	To describe population, medication (previous and concomitant) and clinical outcomes when propofol was used for PS.	Setting: The department of palliative medicine, Akershus University Hospital, Norway.	Dyspnea (*n* = 7), pain (*n* = 6), delirium (*n* = 1).	N/A	- All data collected from patients’ electronic files 1 week before death.
			Population: All terminal patients who received propofol for PS during April 2014 and September 2018.			- Decision to sedate made by two senior consultants after multidisciplinary evaluation.
			Sample: *n* = 14, males 71%, mean age 48 (range 14–87) years. Primary diagnosis: cancer (56%), ALS (21%), intestinal dysfunction (7%), lung fibrosis (7%) and spastic paraplegia (7%).			
Garcia Romo et al., Spain^33^	Retrospective observational study	To describe the efficacy and safety of PS in refractory sedation with propofol using a protocol based on low, incremental dosing.	Setting: The PCU of the Fundación Jiménez Díaz University Hospital, Madrid, Spain.	Psychoexistential suffering (*n* = 6), hyperactive delirium (*n* = 5), multifactorial (*n* = 4), pain (*n* = 3), epileptic status (*n* = 2), dyspnea (*n* = 1), insomnia (*n* = 1).	Depth of sedation measured with RSS.	- All presented risk factors for complicated sedation.
			Population: Terminal patients admitted between March 2018 and February 2023, who were prescribed propofol as a sedative agent.			- Onset of apnea or respiratory depression were monitored in the first 60 min and after dose modification.
			Sample: *n* = 22 (males 64%, mean age 54.36 ± 8.97 years). Primary diagnosis: cancer (90%—lung 27%, colorectal 14%, bile duct 9% and bladder 9%), anoxic encephalopathy (5%) and neurocysticercosis (5%).			
Hedman et al., Sweden^34^	Cross-sectional study	To understand the current practice of PS in Sweden.	Setting: 12 specialized PCU in Skåne, Sweden.	Anxiety (*n* = 14), existential distress (*n* = 11), dyspnea (*n* = 11), pain (*n* = 9), delirium (*n* = 5), other (*n* = 3).	Instructions for assessing depth of sedation: mild (easy to wake); intermittent (periods of being awake); and continuous (no periods of being awake).	- Only 11 PCU participated.
			Population: All consecutive deceased patients between May and October 2016.			- Midazolam used in 94% of patients, propofol in 15%.
			Sample: *n* = 690 (females 50%, mean age 74.5 ± 11.3 years). From these, 53 (8%) received PS. Primary diagnosis: cancer (89%), heart disease (5%), neurological disease (3%) and lung disease (2%).			
Papavasiliou et al., Belgium^35^	Cross-sectional study	To compare and contrast physician-reported practices on CDSUD between GP and MS.	Setting: The Flemish Agency for Care and Health in Flanders, Belgium.	- Drowsiness (*n* = 297), dyspnea (*n* = 133), delirium (*n* = 126), pain (*n* = 64), nausea (*n* = 23)	Symptom intensity in last 24 h rated with NRS.	- Benzodiazepines alone as first line (43 GP, 29 MS) or with opioids (110 GP, 97 MS).
			Population: A stratified random sample of cases of CDSUD between June and November 2007.	- Only one symptom (47%)		- Propofol only reported by MS (16%).
			Sample: *n* = 561 (females 54%, 39% aged between 65 and 79 years). From these, 292 cared by GP and 269 by MS. Primary diagnosis: cancer (37%), cardiovascular disease (25%), respiratory disease (12%), neurological disease (4%) and other (22%).	- Two symptoms (23%)		
				- Three to five symptoms (15%).		
Monreal-Carrillo et al., Mexico^36^	Prospective observational study	To characterize the level of consciousness in patients undergoing PS using BIS monitoring.	Setting: The INCan PCU, Mexico City, Mexico.	Delirium (*n* = 15), pain (*n* = 13), dyspnea (*n* = 6), seizures (*n* = 5), malignant obstruction (*n* = 3), existential distress (*n* = 2), asphyxia (*n* = 1), massive bleeding (*n* = 1).	Levels of sedation evaluated with RSS and BIS at 0, 2, 4, 6, 12, and 24 h.	- More than two symptoms: 90%, namely delirium and pain (45%).
			Population: Inpatients requiring PS between April and November 2015. Patients suffered from advanced cancer (with no further disease-modifying treatment options) and had DNR orders in place.			- Heart rate, blood pressure and temperature were recorded.
			Sample: *n* = 20, females 60%, median age 41 (range 29–71) years. Main cancers: gastrointestinal (20%), breast (15%) and melanoma (15%).			
Schur et al., Austria^37^	Retrospective cohort study	To characterize PS practice at PCU in Austria.	Setting: 23 Austrian PCU.	- Delirium (*n* = 254), existential distress (*n* = 159), dyspnea (*n* = 151), pain (*n* = 99).	N/A	- Midazolam was first line medication (79%).
			Population: All consecutive deceased patients between June 2012 and June 2013.	- Two symptoms (36%).		- Propofol use: 3%.
			Sample: *n* = 2414, females 52%, median age 73 (range 64–82) years. From these, 502 (21%) received PS: CPS (*n* = 356) and IPS (*n* = 119). Primary diagnosis: cancer (84%) and other diseases (15%).	- Three symptoms (8%).		
Ciais et al., France^38^	Before-and-after study	To determine whether propofol is suitable in the care of patients who experience refractory pain at the end of life.	Setting: The PCU of the Centre Hospitalier Universitaire de Nice, France.	Pain (*n* = 10).	Pain ⩾ 3 on NRS or >2 on the Algoplus Scale for patients with inability to speak.	- Propofol stopped immediately after care procedures.
			Population: Terminal patients, admitted during a period of 15 months, who experienced refractory pain.			- Patients monitored until they regained consciousness.
			Sample: *n* = 10, females 70%, mean age 67 (range 44–89) years. Primary diagnosis: cancer (90%) and peripheral artery disease (10%).			

ALS: Amyotrophic Lateral Sclerosis; BIS: Bispectral Index Score; CDSUD: Continuous Deep Sedation Until Death; CPS: Continuous Palliative Sedation; DNR: Do-Not-Resuscitate; GP: General Practitioner; INCan: Instituto Nacional de Cancerologia; IPS: Intermittent Palliative Sedation; MS: Medical Specialist; NRS: Numeric Rating Scale; PC: Palliative Care; PCU: Palliative Care Unit; PS: Palliative Sedation; RASS: Richmond Agitation-Sedation Scale; RSS: Ramsay Sedation Scale.

**Table 2.** Main results of the included studies and syntheses (n = 10).

Authors, Country	Initial and maintenance doses	Treatment effects	Objective of sedation	Protocol	Duration of treatment, survival time	Combination of drugs	Safety and ethical considerations	Key observations
Lundström et al., Sweden^29^	- Initial dose: 0.6–2.0 mg/kg/h (Mean 1.0).	- Good/very good in 20/22 (91%).	- Conscious sedation (*n* = 9).	- Dose titration in steps of 0.5 mg/kg/h.	- Duration of treatment: 2 h to 14 days (Mean 4.9 days).	Propofol + midazolam needed in 6 patients.	Did not monitor blood pressure, heart rate or respiration rate.	- All failed standard treatment (diazepam or midazolam).
	- Maintenance dose: 0.5–6.0 mg/kg/h (Mean 0.90-2.13).	- Moderate in 2/22 (9%).	- Deep sedation (*n* = 12).	- Smaller dose steps required to fine-tune treatment.	- Median survival: 38 days vs usual median survival of 14 days.			- All but 3 died at the unit.
			- 19 patients required continuous treatment until death.					- All but 2 needed strong opioids.
Won et al., Korea^30^	- Initial dose: 0.25–0.5 mg/kg/h.	Propofol alleviated symptoms of 12/16 (75%) midazolam-failure patients.	- PS was successful when RASS ⩽ −1 with controlled symptoms.	Careful titration to reach minimum sedative dose required for symptom control.	- Median duration of treatment: 5 (1.5–11.5) days.	No combination of drugs was used.	-No fatal events noted.	85% Died during hospitalization.
	- Maintenance dose: 0.25–2.0 mg/kg/h.		- Median RASS = −3 for midazolam and propofol.		- Median survival: 28 days. PS vs No PS: 34 vs 25 days (*p* = 0.109).		-Respiratory depression in 10 (11%) midazolam patients and 5 (31%) propofol patients.	
					- Median survival from start of PS: 5 (2–15) days. IPS vs CPS: 6 vs 1 days (*p* < 0.001).		-CPS not found to hasten death.	
Kuragaichi et al., Japan^31^	N/A	Physical (*n* = 151) and mental (*n* = 149) symptomatic relief.	N/A	N/A	There was an opinion that life expectancy improved (*n* = 10).	No combination of drugs was used.	-Most institutions (67%) did not collect written approvals for sedative medications.	- Opioid (87%) and non-opioid analgesics (25%) were prescribed.
							-Propofol without approval in 14% of cases.	- 91% were prescribed analgesics and/or sedatives for hospitalization, whilst 9% were prescribed also for home use.
Fredheim et al., Norway^32^	Maintenance dose: 20–340 mg/h (Mean 137.7).	All patients achieved adequate symptom control.	-3 Patients had spontaneous awakenings.	-No formal protocol.	Survival from start of PS: <24 h (*n* = 9), 24–48 h (*n* = 4) and 5 days (*n* = 1).	No combination of drugs was used.	-Respiratory depression (*n* = 1), airway obstruction (*n* = 1).	- 6 Patients failed previous treatment with midazolam.
			-Sedation and symptom control were re-established and maintained after further titration.	-Clinical effects and dose adjustments evaluated bedside by nurses and physicians.			-No circulatory collapse noted.	- All patients received opioids.
							-Measures against 1 patient’s will to end life-prolonging therapy were implemented.	
Garcia Romo et al., Spain^33^	- Initial dose: 20–30 mg/h.	All patients achieved satisfactory levels of sedation with symptom control.	Most required profound sedation (RSS levels 5 or 6).	Propofol started at low doses (20–30 mg/h) and was increased (20 mg/h each 5 min) until minimal level of sedation for symptom control.	Median survival from start of PS: 26 (4.1–39.5) h.	77% Required other sedatives, such as midazolam and/or neuroleptics.	No cases of apnea or death during induction were recorded.	All previously failed sedation with other drugs.
	- Median maintenance dose: 210 mg/h.							
Hedman et al., Sweden^34^	Median maintenance dose: 70 mg/h.	Adequate symptom control in 73% at 24 h of treatment and 52% in the last 24 h of life.	- 42% died in continuous sedation.	N/A	Duration of treatment: 2–504 h (Median 36).	Propofol + midazolam used in 11% of patients.	16% Of patients without decision to sedate died deeply unconscious.	- 72% of PS and 43% of No PS patients died in hospital.
			- None were awake.					- 23% of PS and 42% of No PS patients died at home.
Papavasiliou et al., Belgium^35^	N/A	Symptoms (GP vs MS):	No significant differences in consciousness level between groups.	N/A	Duration of treatment (GP vs MS):	Combination of sedatives (GP vs MS): 1% vs 7% (*p* = 0.004).	- Explicit life-shortening intentions in both groups (3%).	3–5 symptoms (GP vs MS): 22% vs 9% (*p* < 0.001).
		-Pain (3.07 vs 1.60; *p* < 0.001).			- 0–24 h (25% vs 34%; *p* = 0.049).		- Sedation without patient or family consent (GP vs MS): 5% vs 28% (*p* < 0.001).	
		-Delirium (4.97 vs 2.49; *p* < 0.001).			- 1–7 days (68% vs 56%; *p* = 0.049).			
		-Dyspnea (4.31 vs 3.50; *p* = 0.003).			- 1–2 weeks (6% vs 7%; *p* = 0.049).			
		-Nausea (1.87 vs 1.05; *p* = 0.016).			- Over 2 weeks (1% vs 4%; *p* = 0.049).			
Monreal-Carrillo et al., Mexico^36^	-Initial dose: 0.16 mg/kg/h.	4 patients had RSS = 4–6 and BIS < 60 at 24 h and did not require dose escalation.	- Time and wakefulness (RSS vs BIS): 0 h (70% vs 95%), 4 h (31% vs 56%), 6 h (27% vs 53%), 24 h (22% vs 33%).	- MIDAS PS protocol (propofol + midazolam).	- Median duration of treatment: 24.5 (6–46) h.	All patients received propofol + midazolam.	BIS reading was informative even as death approached.	- Other drugs (opioids, antipsychotics) were continued during sedation.
	-Maintenance dose: 0.16–1.3 mg/kg/h.		- At each point, a small number of patients with RSS = 4–6 were awake by BIS > 60.	- Dosage increased if RSS = 1–3 or BIS > 60.	- Median survival: 19 h.			- A tendency was observed toward the increase of RSS as BIS decreased (moderate correlation; rho = −0.68 to −0.46).
Schur et al., Austria^37^	No significant increase from initial to maintenance doses.	Symptom control in 88% of patients with only one drug.	N/A	N/A	- Median duration of treatment: 48 (10–72) h.	11% Required more than one drug.	Sedation did not exert a negative impact on patients’ survival.	- 94 patients (4%) died on the day of admission.
					- Median survival: 9 (4–20) days. PS vs No PS: 10 vs 9 days (*p* = 0.491).			- Sedated patients received more opioids. PS vs No PS: 96% vs 90% (*p* < 0.001).
					- No difference in CPS vs IPS (*p* = 0.259).			
Ciais et al., France^38^	-Initial dose: 40–100 mg (Mean 67).	Care delivered without pain in 9/10.	- Targeted depth of sedation: absence of verbal contact.	- Propofol started at 20 mg/kg/h until targeted depth of sedation.	- Duration of treatment: 7–32 min (Mean 13).	No combination of drugs was used.	One patient experienced prolonged apnea requiring interruption of care procedures.	- All patients received opioids.
	-Maintenance dose: 28–105 mg (Mean 69).		- All patients reached desired sedation levels.	- Then, dosage was reduced to 6 mg/kg/h.	- Mean survival: 15 days.			- Death rate was 80%.
					- Duration of induction: 2–8 min (Mean 4).			
					- Time to regain consciousness: 7–18 min (Mean 11.5).			

BIS: Bispectral Index Score; CPS: Continuous Palliative Sedation; GP: General Practitioner; IPS: Intermittent Palliative Sedation; MIDAS: Intensive Management of Pain, Anxiety and Distress; MS: Medical Specialist; PS: Palliative Sedation; RASS: Richmond Agitation-Sedation Scale; RSS: Ramsay Sedation Scale.

### Certainty assessment

The certainty of evidence for each outcome was assessed using the Grading of Recommendations Assessment, Development and Evaluation (GRADE) approach.^15^ Certainty ratings were determined across the domains of risk of bias, inconsistency, indirectness, imprecision, and publication bias. Evidence from observational studies was initially rated as low certainty and downgraded where serious limitations were identified.

The GRADE assessment was conducted independently by the two reviewers, with disagreements resolved through discussion and consensus.

Confidence in qualitative findings was not assessed, as no formal qualitative synthesis was conducted.

## Results

### Study selection

A total of 91 articles were identified through database searching. After removal of duplicates, 57 records remained. During title and abstract screening, 34 articles were excluded. The most common reasons were: studies not addressing palliative sedation (e.g. articles on general anesthesia, perioperative care, or propofol in non-palliative indications); patient populations outside the scope (e.g. pediatric, non-terminal illness, or experimental animal studies); articles without extractable clinical data (e.g. commentaries, case reports of a single patient, and articles without extractable clinical data (e.g. commentaries, case reports of a single patient, or conference abstracts).

**Figure 1.**
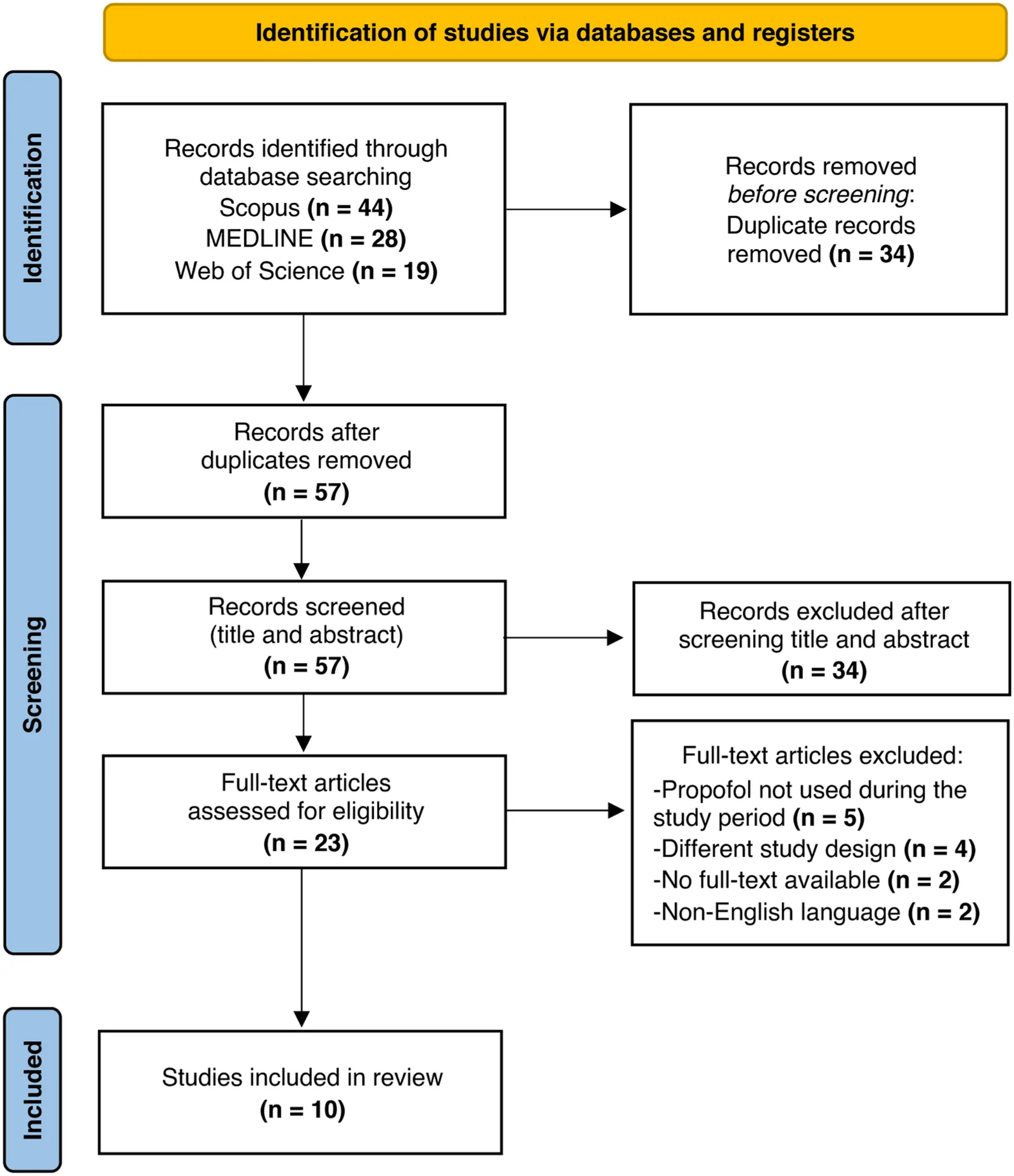
Flow diagram of the study selection process.

Twenty-three full-text articles were assessed for eligibility; of these, 13 were excluded due to: propofol not used in the study period (*n* = 5)^16–20^; study design not meeting eligibility criteria (*n* = 4, including secondary analysis, physicians’ opinion/practice survey, nationwide questionnaire survey, and clinicians’ experiences/attitudes study)^21–24^; no full-text available (*n* = 2)^25,26^; and publication in languages other than English (*n* = 2).^27,28^ The rationale for exclusion of full-text articles is presented in Table 3. Finally, 10 studies met the criteria for inclusion in this review.^29–38^ A flow diagram of the study selection process is presented in Figure 1.

**Table 3.** Rationale for exclusion of full-text articles (n = 13).

Authors, Country	Reason for exclusion
Schildmann et al., Germany^16^	Propofol not used in the study period
Díez-Manglano et al., Spain^17^	Propofol not used in the study period
Schildmann et al., Germany^18^	Propofol not used in the study period
Schildmann et al., Germany^19^	Propofol not used in the study period
Maeda et al., Japan^20^	Propofol not used in the study period
Morita et al., Japan^21^	Secondary analysis
Heijltjes et al., Netherlands^22^	Physicians’ opinion and practice
Kurozumi et al., Japan^23^	Nationwide questionnaire survey
Maiser et al., United States of America^24^	Clinicians’ experiences and attitudes
Prampart et al., France^25^	No full-text available
Jacquin et al., Monaco^26^	No full-text available
Olivier et al., France^27^	Other languages
Hopprich et al., Germany^28^	Other languages

### Study characteristics

The characteristics of the individual studies are summarized in Table 1.

Ten studies were included: three cross-sectional studies (two analytic and one descriptive)^31,34,35^; two cohort studies (one retrospective and one prospective)^30,37^; three other observational studies (two retrospective and one prospective)^32,33,36^; one case series^29^; and one before-and-after study.^38^ Most studies were conducted in Europe, including Sweden,^29,34^ Norway,^32^ Spain,^33^ Belgium,^35^ Austria,^37^ and France.^38^ Additional studies were conducted in Asia (Korea^30^ and Japan),^31^ as well as in America (Mexico).^36^ The 10 studies comprised a total of 4072 patients (28.3% received palliative sedation), and 544 institutions. Nine studies evaluated patients with cancer, either exclusively,^29,30,36^ or alongside other diagnoses.^32–35,37,38^ One study focused solely on patients with heart failure.^31^ All studies took place in palliative care units or hospice units, except for one study,^31^ which focused on hospitalized patients. One study evaluated short-term propofol sedation during painful care procedures in terminally ill patients receiving palliative care.^38^ Although this differed from conventional continuous or intermittent palliative sedation, it involved intentional reduction of consciousness to relieve refractory suffering and was therefore considered eligible for inclusion.

Qualitative findings, obtained through interviews, were reported in two studies.^31,32^

### Risk of bias in studies

The risk of bias for non-randomized studies (*n* = 9) is presented in Table 4. All studies, except for Kuragaichi et al.^31^ and Ciais et al.,^38^ were free of serious risk of bias across all domains. These two studies were included, in spite of their elevated risk of bias, because they evaluated specific populations not otherwise represented: non-oncologic patients,^31^ and short-term sedation.^38^ The following assessment is presented by domain of bias risk:

**Table 4.** Risk of bias for non-randomized studies of interventions (n = 9).

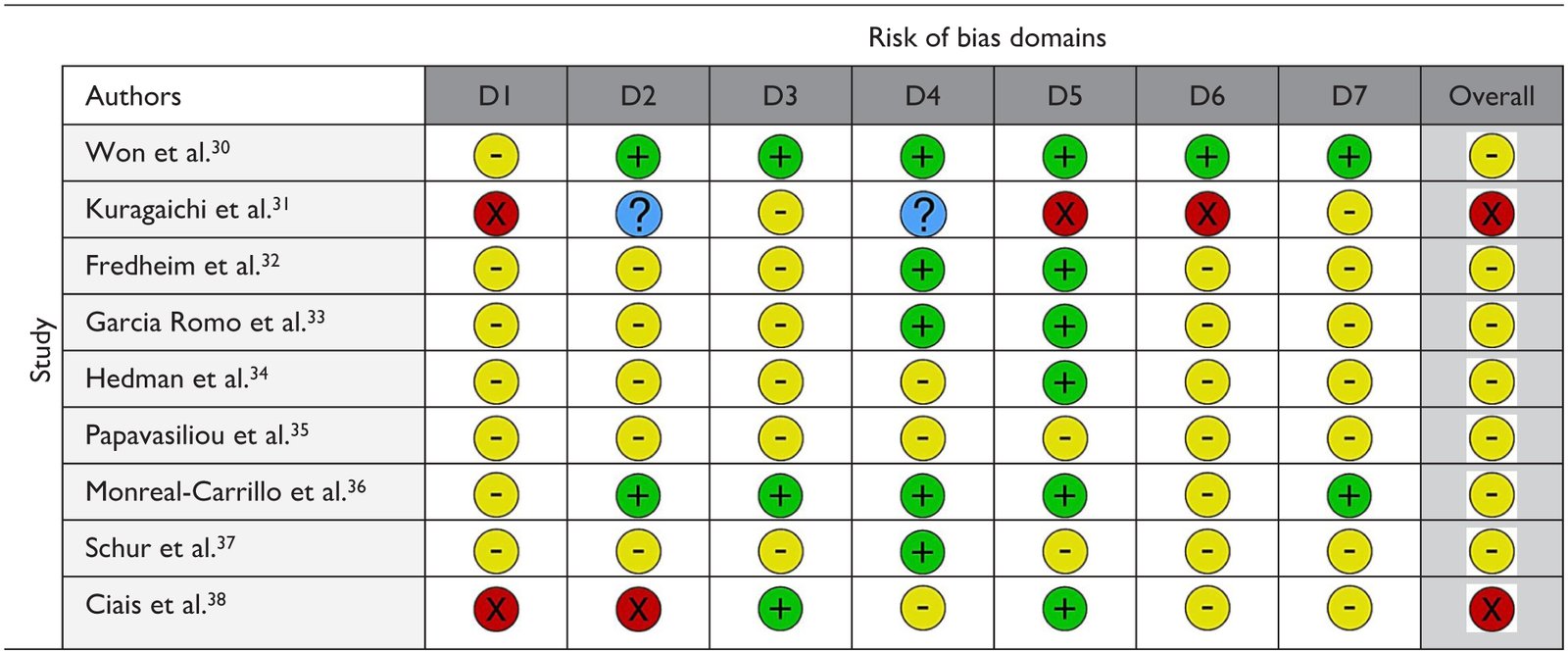

Domains: **D1:** Bias due to confounding; **D2:** Bias due to selection of participants; **D3:** Bias in classification of interventions; **D4:** Bias due to deviations from intended interventions; **D5:** Bias due to missing data; **D6:** Bias in measurement of outcomes; **D7:** Bias in selection of the reported result.

**Judgment:**

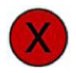
 Serious 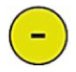 Moderate.

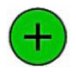
 Low 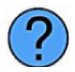 No information.

#### Confounding

Most studies were at moderate risk of bias due to confounding. Kuragaichi et al.^31^ was judged at serious risk, as institutional characteristics were not appropriately controlled. Ciais et al.^38^ also carried serious risk, given the considerable heterogeneity of the participating sample, which was difficult to control.

#### Participant selection

Bias from participant selection was moderate in the majority of studies,^32–35,37^ mainly because the start of follow-up and the intervention did not coincide (retrospective designs). Won et al.^30^ and Monreal-Carrillo et al.^36^ were at low risk, as all eligible participants were included and the timing of follow-up and intervention coincided. Ciais et al.^38^ was at serious risk, since selection was related to both intervention and outcome and could not be adjusted for. Kuragaichi et al.^31^ did not provide sufficient information on participant selection.

#### Classification of interventions

Intervention status was clearly defined in all studies. Risk of bias was therefore low in Won et al.,^30^ Monreal-Carrillo et al.,^36^ and Ciais et al.,^38^ and moderate in the remaining studies where some aspects of the intervention had been defined retrospectively.^31–35,37^

#### Deviations from intended interventions

Minor deviations occurred in most studies but were unlikely to have influenced outcomes. Thus, risk of bias was considered low,^30,32,33,36,37^ or moderate.^34,35,38^ Kuragaichi et al.^31^ did not provide information on this domain.

#### Missing data

Risk of bias due to missing data was low in most studies.^30,32–34,36,38^ In two studies,^35,37^ missing data were minimal and risk was therefore moderate. In Kuragaichi et al.,^31^ however, substantial missing data led to a serious risk rating.

Outcome measurement was considered moderately biased in most studies,^32–38^ since assessment could have been minimally influenced by knowledge of the intervention. Won et al.^30^ was at low risk because outcomes were unlikely to be affected by such knowledge and measurement procedures were consistent across groups. Kuragaichi et al.,^31^ which relied on subjective outcome measures, was rated at serious risk.

#### Selective reporting

For most studies, risk of selective reporting was judged moderate.^31–35,37,38^ In Won et al.,^30^ and Monreal-Carrillo et al.,^36^ risk was low, as all reported results corresponded to prespecified outcomes, analyses, and subcohorts.

#### Case series

In the study by Lundström et al.^29^ inclusion criteria were explicit, the intervention was clearly defined and reproducible, and participant inclusion was consecutive and complete. The main limitation was outcome measurement, which relied on observer-reported subjective scales and thus carried moderate risk of bias.

### Results of individual studies

#### Quantitative outcomes

Table 2 summarizes the quantitative outcomes reported in the included studies.

#### Qualitative outcomes

Qualitative findings were reported in two studies that incorporated interviews alongside quantitative assessments.^31,32^ Patients and, where available, caregivers emphasized the perceived value of palliative sedation in enhancing dignity at the end of life and alleviating physical and psychoexistential suffering. These findings were descriptive and supportive in nature.

### Results of syntheses

Most studies focused on elderly patients with terminal oncologic disease. All studies took place in palliative care units or hospice units, except for one study,^31^ which focused on hospitalized patients.

Overall risk of bias was moderate for eight studies,^29,30,32–37^ and serious for two studies.^31,38^ Studies with increased risk of bias had potential limitations related to handling of missing data, length of follow-up, whether confounders were assessed with reliable measures, and whether outcomes were prespecified.

#### Symptom control

All 10 studies assessed symptom control. Results varied from 73%,^34^ to 100%.^32,33^

In all studies, more than one refractory symptom was present, except in one study where pain was the sole refractory symptom and constituted the indication for palliative sedation.^38^

The most frequent indications for palliative sedation were delirium in three studies,^30,36,37^ anxiety in two studies,^29,34^ dyspnea in two studies (one with moderate risk of bias, the other with serious risk of bias),^31,32^ psychoexistential suffering in one study,^33^ and pain in one serious risk of bias study.^38^ However, the methods used to assess symptom control varied across studies, and in several cases improvement was inferred indirectly from sedation depth or clinician judgment rather than standardized symptom measurement tools, which limits comparability across studies.

#### Consciousness level

Eight studies assessed consciousness level.^29,30,32–36,38^ The targeted depth of sedation was achieved in 52%,^34^ to 100% of patients.^32,33,38^ The objective of sedation was^39^: moderate sedation in six studies (five with moderate risk of bias, one with serious risk of bias) ^29,30,32,34,36,38^; and deep sedation in two studies.^33,35^

#### Survival time

Eight studies assessed survival time.^29–33,36–38^ Results varied from 19 h^36^ to 38 days.^29^ In six studies (five with moderate risk of bias, one with serious risk of bias) most patients died during hospitalization.^29,30,32,34,37,38^ Two studies evaluated the difference in survival time between sedated and non-sedated patients,^30,37^ concluding an increase in survival with palliative sedation of 1 day (*p* = 0.491)^37^ to 9 days (*p* = 0.109).^30^ These findings should be interpreted cautiously given the observational nature of the included studies and potential differences in baseline patient characteristics.

#### Propofol utilization

Propofol was used in monotherapy in four studies (two with moderate risk of bias, two with serious risk of bias),^30–32,38^ and in combination with other sedatives in six studies.^29,33–37^ Five studies administered propofol according to a predefined dosing regimen, specifying initial and maintenance doses.^29,30,33,36,38^ Initial doses ranged from 0.16^36^ to 2.0 mg/kg/h,^29^ and maintenance doses from 0.16^36^ to 6.0 mg/kg/h.^29^

#### Safety considerations

The primary safety concern reported was respiratory depression, in three studies (two with moderate risk of bias, one with serious risk of bias).^30,32,38^ Frequencies ranged from 7%^32^ to 31%.^30^ These studies used propofol in monotherapy. However, reporting of the clinical consequences of respiratory depression was inconsistent, and it was not always clear whether these events required specific intervention or represented expected physiological effects associated with deep sedation.

#### Ethical implications

Key ethical concerns included sedation without patient or family consent, reported in four studies (three with moderate risk of bias, one with serious risk of bias),^31,32,34,35^ with individual values ranging from 5% to 28% (*p* < 0.001).^35^ One study also reported explicit life-shortening intentions in 3% of all cases (*p* < 0.001).^35^ These findings highlight areas of potential ethical concern requiring careful contextual interpretation.

### Certainty of evidence

Using the GRADE approach, certainty of evidence was low for achievement of target sedation depth and very low for symptom control, survival time, and respiratory depression. Downgrading was mainly due to the observational design of the included studies, overall serious risk of bias, heterogeneity in outcome assessment, and imprecision. Detailed assessments are presented in Table 5.

**Table 5.** Propofol for palliative sedation: GRADE evidence profile (n = 10).

Outcome	Contributing studies	Participants	Main findings	Risk of bias^a^; Inconsistency^b^	Indirectness^c^	Imprecision^d^	Certainty of evidence
Symptom control	Lundström et al.,^29^ Won et al.,^30^ Kuragaichi et al.,^31^ Fredheim et al.,^32^ Garcia Romo et al.,^33^ Hedman et al.,^34^ Papavasiliou et al.,^35^ Monreal-Carrillo et al.,^36^ Schur et al.,^37^ Ciais et al.^38^	1306 Patient-level PS/PE cases	Reported improvement ranged from 73% to 100%; assessment methods varied and were often indirect.	Serious	Serious	Serious	VERY LOW (⊕○○○)
Target sedation depth	Won et al.,^30^ Kuragaichi et al.,^31^ Fredheim et al.,^32^ Garcia Romo et al.,^33^ Hedman et al.,^34^ Monreal-Carrillo et al.,^36^ Schur et al.,^37^ Ciais et al.^38^	710 Patient-level PS/PE cases	Targeted sedation depth achieved in 52%–100% of cases.	Serious	Not serious	Not serious	LOW (⊕⊕○○)
Survival time	Won et al.,^30^ Kuragaichi et al.,^31^ Fredheim et al.,^32^ Garcia Romo et al.,^33^ Hedman et al.,^34^ Monreal-Carrillo et al.,^36^ Schur et al.,^37^ Ciais et al.^38^	710 Patient-level PS/PE cases	Reported survival ranged from 19 h to 38 days; no reliable causal inference possible.	Serious	Very serious	Serious	VERY LOW (⊕○○○)
Respiratory depression	Won et al.,^30^ Kuragaichi et al.,^31^ Ciais et al.^38^	99 Patient-level PS/PE cases	Reported in 7%–31% of cases; clinical consequences inconsistently described.	Serious	Serious	Serious	VERY LOW (⊕○○○)

GRADE: Grading of Recommendations Assessment, Development and Evaluation; PS/PE: palliative sedation/propofol exposed.

Note. Kuragaichi et al.^31^ represents an institutional survey (544 institutions) rather than a patient-level cohort and was therefore included as a contributing study but excluded from pooled patient-level population estimates.

a Downgraded for risk of bias due to predominance of retrospective observational designs, moderate-to-serious risk of bias in several studies, potential confounding, and reliance on clinician-reported outcomes.

b Downgraded for inconsistency due to substantial variability in reported outcome definitions, clinical indications, sedation practices, and observed effect ranges across studies.

c Downgraded for indirectness due to heterogeneity in patient populations, sedation indications, and outcome measurement methods, including reliance on proxy indicators such as sedation depth rather than standardized symptom scales.

d Downgraded for imprecision due to small sample sizes in contributing studies and limited reporting of outcome-specific events.

## Discussion

### Executive summary

This systematic review (*n* = 10) evaluated the use of propofol in palliative sedation. The findings suggest that propofol can achieve symptom control (73%–100%) and target sedation depth (52%–100%), either as monotherapy or in combination with other sedatives. Reported survival times varied widely (19 h–38 days). Respiratory depression was the primary safety concern, while identified ethical issues included sedation without consent and explicit life-shortening intentions. Despite its potential role as a palliative sedation agent, the lack of conclusive evidence on feasibility and ethical acceptability underscores the need for further research and clearer clinical guidance.

The available evidence should be considered largely hypothesis-generating, given the predominance of observational study designs, heterogeneity in sedation practices, and variability in outcome measurement across studies. Certainty of evidence was low to very low across outcomes, reflecting methodological limitations and the limited ability of available data to support causal inference, particularly for survival and safety outcomes.

### General interpretation of the results

#### Symptom control

Palliative sedation effects were measured by focusing on symptom control, consciousness level, opinions of healthcare professionals and relatives, or survival outcomes. In several studies, symptom relief was inferred from achieved sedation depth rather than direct symptom measurement, raising the possibility of circular reasoning and limiting the interpretability of reported clinical outcomes.

All studies included in this review reported the presence of refractory symptoms at the end of life as an essential requirement for palliative sedation. The majority of these symptoms were delirium, dyspnea, and pain. In Papavasiliou et al.^35^ refractory symptoms were reported as a primary driver for sedation in over 70% of cases, although it was difficult to determine the predominant symptoms requiring palliative sedation, since multiple refractory symptoms were present. Schur et al.^37^ noted similar trends. Most included studies analyzed time to sedation as an indirect marker of symptom control, with shorter time to sedation indicating quicker symptom relief.^29–37^

Additional studies have also detailed the occurrence and management of refractory symptoms in end-of-life care. Patel et al.^3^ reported that refractory symptoms, particularly dyspnea and agitation, frequently persist in terminal patients despite optimal pharmacological interventions, often necessitating palliative sedation. Similarly, in their systematic review, Maltoni et al.^40^ emphasized that refractory symptoms, especially delirium and dyspnea, were prevalent in over 50% of terminal cancer patients, leading to the frequent use of sedation protocols. Furthermore, Bruinsma et al.^41^ highlighted the complexity of diagnosing refractory symptoms, noting that symptoms such as existential distress and severe anxiety often challenge standard palliative care measures and require multidisciplinary approaches including sedation.

Most studies did not specify the methods used for assessment of refractory symptoms,^29–34,36,37^ limiting cross study comparisons. In one study,^38^ pain was evaluated using the Numeric Rating Scale or the Algoplus Scale for patients with inability to communicate verbally,^42^ allowing for better symptom discrimination. Anxiety and/or psychoexistential suffering were also frequently reported, however, these lacked a clear definition or specific assessment tools.

Studies such as those by de Graeff and Dean,^43^ and Claessens et al.^44^ emphasized the importance of incorporating patient-reported outcomes and interdisciplinary team assessments. The former advocated that symptom assessment should involve continuous dialogue between patients, families and multidisciplinary teams to evaluate symptom severity and the impact of interventions,^43^ while the latter focused on the role of palliative care consultations, suggesting that interdisciplinary teams, including psychologists, social workers and spiritual care providers, enhance the understanding of refractory symptoms by integrating both medical and psychosocial perspectives.^44^

Many of the included studies were conducted within palliative care services, where medical specialists are accustomed to managing complex clinical situations. This is highlighted in the study by Papavasiliou et al.^35^ which found that mean symptom intensity was consistently higher in cases treated by general practitioners. However, there appears to be a lack of further studies directly addressing differences in the assessment or management of refractory symptoms across medical specialties in the context of palliative sedation.

### Consciousness level

The levels of sedation induced by propofol varied across studies, and this can be attributed to distinct infusion rules, variations in dosage and titration scheme, the use of adjuvant medications, as well as to different methods of assessing sedation levels (e.g. Richmond Agitation-Sedation Scale, Ramsay Sedation Scale, Bispectral Index Score).

The EAPC guidelines recommend combining subjective caregiver assessments with objective tools to monitor sedation efficacy and patient comfort.^4,45^ They also emphasizes the principle of proportionality in palliative sedation practice.^4^ First, in terms of depth of sedation, sedative medications should be titrated to the lowest dosage that provides adequate relief of suffering and maintains the most interactive function.^4^ Second, in terms of timing, intermittent sedation should be sought whenever possible.^4^ Regular interdisciplinary team reviews are also recommended to optimize sedation protocols based on continuous assessments, emphasizing patient-centered care and ethical practice.^45^ The American Academy of Hospice and Palliative Medicine, and the Hospice and Palliative Nurses Association endorsed similar individualized monitoring approaches.^46^

Some technical approaches, such as the Bispectral Index Score,^36,47,48^ are emerging to evaluate consciousness levels beyond a patient’s ability to respond.^47,49^ While this score was used in one included study,^36^ its reliability in clinical practice requires further validation. Additionally, the need for specific equipment and the wide range of values in deeply sedated patients makes its use in routine clinical practice questionable.^50^

### Survival time

Although survival analysis is relevant when assessing potential adverse effects of sedation, the primary objective of palliative sedation remains symptom control and relief of refractory suffering. Survival varied considerably across the included studies. Schur et al.^37^ reported no significant difference in survival time between sedated and non-sedated patients. Other studies reported slight differences in survival duration; however, these findings were statistically insignificant and derived from observational designs.^29–31^

Two studies evaluated intermittent versus continuous palliative sedation.^30,37^ In one study,^37^ no difference in survival was observed. In the other,^30^ median survival in the intermittent sedation group was longer than in the continuous sedation group. The authors attributed this difference to baseline clinical status, with patients receiving continuous sedation generally presenting with more advanced disease and poorer prognostic status. Observational comparisons between sedated and non-sedated patients remain highly susceptible to confounding by indication, as patients receiving sedation typically have more advanced disease or more severe symptoms. Consequently, causal inference regarding the effect of propofol on survival cannot be established.

Previous systematic reviews, including that of Maltoni et al.,^40^ have similarly reported no clear evidence that palliative sedation shortens survival, although these findings are largely based on observational data.^51–54^ The European Society for Medical Oncology (ESMO) Clinical Practice Guidelines state that palliative sedation, when appropriately administered, does not shorten survival time.^5^ The revised EAPC framework emphasizes that the primary intention of palliative sedation is relief of refractory suffering rather than life-shortening, reinforcing its ethical acceptability when applied proportionally and according to established safeguards.^4^

Overall, the available evidence remains insufficient to determine the precise impact of propofol-based palliative sedation on survival. Further prospective studies with careful adjustment for prognostic variables are needed to clarify this relationship.

### Propofol utilization

Propofol was used in combination with other sedatives in six of the included studies,^29,33–37^ while four studies reported propofol monotherapy.^30–32,38^ Reported dosing regimens were generally comparable across studies, although precise dosage details were not consistently specified, limiting comparability of results. In this review, initial infusion rates ranged from 0.16 to 2.0 mg/kg/h,^29,36^ with maintenance doses up to 6.0 mg/kg/h.^29^ Considerable heterogeneity in dosing strategies further limits direct comparison of outcomes and underscores the need for standardized reporting. The dosing ranges observed were broadly consistent with examples summarized in Supplemental Table 2, illustrating substantial variability in induction and maintenance regimens across studies.^9,55–60^

Propofol monotherapy can achieve adequate sedation but lacks intrinsic analgesic properties, which may limit its suitability in patients with significant pain.^32^ Higher doses required to achieve deep sedation may also increase the risk of adverse effects such as hypotension and respiratory depression.^32^ Conversely, combination therapy may provide synergistic effects, allowing lower doses of individual agents and potentially reducing adverse effects.^33^

In half of the included studies,^30,31,34,35,37^ propofol was introduced as a second- or third-line option and administered to a minority of patients. This pattern reflects its role primarily as a rescue therapy and limits the strength of conclusions regarding routine use. Nevertheless, inclusion of these studies allowed broader characterization of clinical practice patterns.

The requirement for intravenous administration represents an important practical consideration.^4,32,61,62^ Unlike many standard sedatives that can be delivered subcutaneously, propofol requires intravenous access and close monitoring, which may not be routinely available or preferred in all palliative care settings. In several healthcare systems, particularly hospice and community-based services, subcutaneous administration is commonly favored for comfort and practicality. Consequently, propofol use may be restricted to specialized settings with appropriate expertise and monitoring capacity.^4,32,61,62^

Current clinical guidance, including recommendations from the ESMO and the EAPC, emphasizes individualized dose titration and continuous monitoring to ensure effective symptom relief while avoiding excessive sedation.^4,5^ Morita et al.^63^ further highlighted the importance of standardized assessment tools, combined with regular interdisciplinary review of patient status, to support safe and ethically sound sedation practices.

These practical and clinical considerations highlight the need for clearer definitions of clinical scenarios, standardized sedation protocols, and targeted research addressing specific clinical questions.^64^ Although propofol has been successfully used in selected refractory cases, the feasibility and acceptability of broader implementation remain uncertain, particularly in settings with limited infrastructure, monitoring capacity, or specialized expertise.

### Safety considerations

Propofol is associated with clinically important adverse effects, including respiratory depression, hypotension, and bradycardia.^5–8^ Among the included studies, respiratory depression was the most frequently reported adverse effect, with prevalence ranging from 7% to 31%.^30,32^ This variability may reflect differences in dosing regimens, patient characteristics, and sedation depth. However, the clinical significance of these events should be interpreted cautiously, as studies did not consistently distinguish between expected physiological changes during deep sedation and events requiring active intervention.

A rare but serious complication, propofol infusion syndrome, has been associated with prolonged high-dose infusions, particularly in critically ill patients, and may manifest as metabolic acidosis, rhabdomyolysis, hyperlipidemia, and cardiac failure.^65^ To reduce this risk, current recommendations emphasize the use of the lowest effective dose for the shortest duration necessary.^65^

Although short-term use of propofol is generally advised to minimize drug accumulation and adverse effects, the duration of administration varied across the included studies and was not consistently reported. Where available, reported durations ranged from brief rescue use to continuous sedation until death, which has important implications for safety monitoring and risk management.

Overall, available safety data remain limited, and further research is needed to better characterize the respiratory and long-term safety profile of propofol in palliative sedation.

### Ethical implications

Consent represents a central ethical safeguard in palliative sedation. In several included studies,^31,32,34,35^ sedation was initiated without prior patient or family consent, contradicting established guideline recommendations and potentially reflecting deficiencies in documentation or decision-making processes.^4,5,43,45^ Within contemporary ethical frameworks, informed consent and shared decision-making are considered core requirements to respect patient autonomy and ensure proportional use of sedation.^5,43,45^ The revised EAPC framework further emphasizes these processes as essential safeguards for ethically appropriate practice.^4^

Ethical decision-making in palliative sedation extends beyond formal consent to relational processes involving dialogue, responsiveness, and mutual respect among clinicians, patients, and families.^66^ In situations where patients lack decision-making capacity and family members are unavailable, clinicians may initiate sedation based on the patient’s best interests.^67,68^ Such circumstances highlight tensions between autonomy and the principles of beneficence and non-maleficence, particularly when urgent symptom control is required. Shared decision-making involving patients and next of kin remains feasible in most cases, although reliance on advance directives or clinician judgment may occasionally be necessary.^32^ The reported absence of documented consent should therefore be interpreted cautiously, as lack of documentation does not necessarily indicate absence of consent in practice. Nonetheless, clear documentation remains a fundamental safeguard emphasized in the EAPC framework.^4^

One included study reported instances of palliative sedation with explicit life-shortening intentions.^35^ This finding is concerning because it blurs the conceptual boundary between palliative sedation and euthanasia. The ethical legitimacy of palliative sedation rests on the intention to relieve refractory suffering rather than to hasten death, a distinction consistently emphasized across contemporary frameworks and reinforced by the principle of proportionality.^4,69^ Reports suggesting life-shortening intention therefore represent deviations from accepted ethical standards and require careful interpretation within appropriate clinical governance structures.

The distinction between proportional sedation and deep continuous sedation carries additional ethical implications. Proportionality requires titration of sedation to the minimum level necessary to relieve suffering, whereas deep continuous sedation represents a more intensive intervention requiring explicit justification, reassessment, and documentation.^4,69,70^ Recommendations further emphasize that sedation depth and duration should be individualized and regularly reviewed to ensure ethical appropriateness.^70^

Many ethical concerns identified in this review—particularly those related to consent, decision-making capacity, and concerns about potential life-shortening—are not specific to propofol but reflect broader challenges in palliative sedation practice. However, certain pharmacological characteristics of propofol, including rapid onset, intravenous administration requirements, and potential to induce deep sedation, may increase the importance of careful monitoring, proportional titration, and documentation.^32^

The included studies remain largely hypothesis-generating, and formal evaluation of ethical practices remains limited. Although randomized controlled trials could theoretically strengthen the evidence base, such studies present significant ethical and practical challenges in vulnerable end-of-life populations. Carefully designed prospective observational studies incorporating structured ethical evaluation may therefore represent more feasible approaches.

Taken together, these findings underscore the importance of structured ethical governance, multidisciplinary decision-making, and adherence to consensus frameworks such as the revised EAPC recommendations.^4^ Recent guideline developments further emphasize specialist involvement, clear communication, and professional competence as safeguards against ethically inappropriate sedation practices.^70^ Future research should incorporate systematic reporting of ethical processes alongside clinical outcomes to ensure transparency, proportionality, and patient-centered decision-making.

### Comparison with prior reviews

The findings of this review are broadly consistent with previous literature addressing palliative sedation and the use of propofol in refractory cases. The early systematic review by McWilliams et al.^6^ identified only four studies, all case reports or small case series, reflecting the limited evidence available at that time. Although more recent studies have been published, the present review demonstrates continued reliance on observational designs and heterogeneous reporting, indicating that many of the methodological limitations identified previously persist. Consistent with broader reviews of palliative sedation, including those by Arantzamendi et al.,^2^ midazolam remains the primary sedative, with propofol typically reserved for refractory symptoms. Narrative and case-based literature, including the work of Patel et al.,^3^ has emphasized the complexity of decision-making surrounding palliative sedation, highlighting the need for multidisciplinary planning, clear documentation, and careful communication with patients and families. These considerations align with the ethical and governance challenges identified in the present review. Bodnar highlighted several pharmacological advantages of propofol, including rapid onset and short duration of action, but also emphasized the need for experienced teams and appropriate monitoring due to potential respiratory and hemodynamic effects.^9^

The present review builds upon prior work by systematically synthesizing propofol-specific evidence across multiple clinical outcomes, highlighting ongoing variability in dosing strategies, monitoring practices, and feasibility considerations that warrant further standardized investigation.

### Strengths and limitations

A key strength of this review is that it provides an updated and comprehensive synthesis of propofol use for palliative sedation, an area that remains relatively underexplored. A rigorous search strategy across multiple databases and adherence to PRISMA guidelines ensured systematic study selection and critical appraisal of methodological quality. The review integrates clinical, safety, and ethical perspectives, offering structured evaluation of outcomes such as symptom control, sedation depth, and survival, while addressing governance considerations central to palliative sedation practice.

Several limitations should be acknowledged. Restricting the search to English-language publications may have excluded relevant studies. In addition, citation tracking and reference list screening were not performed, and controlled vocabulary (e.g. MeSH terms) was not systematically used. These factors may have reduced search sensitivity and contributed to omission of potentially relevant studies, although use of multiple databases and predefined search terms was intended to mitigate this risk.

Considerable heterogeneity across study design, patient populations, clinical settings, sedation indications, and propofol administration strategies limited comparability and precluded quantitative synthesis. In addition, one included study evaluated short-term procedural sedation during painful care rather than conventional palliative sedation, potentially contributing to conceptual heterogeneity.^38^ Together with the predominance of observational designs and non-standardized dosing protocols, this introduced risks of bias, including selection bias and confounding. The absence of randomized controlled trials further limits causal inference regarding clinical outcomes.

Most included studies involved patients with cancer and were conducted in specialized palliative care settings, which may limit transferability to patients with non-malignant conditions or other care environments. Exclusion of intensive care unit studies, although intended to preserve conceptual consistency, may have resulted in omission of clinically relevant transition-to-palliative cases, thereby limiting generalizability.

Additional methodological limitations include exclusion of some studies due to inaccessible full texts without author contact, and the absence of formal inter-reviewer agreement statistics. Although disagreements were resolved through consensus, lack of quantitative agreement metrics limits assessment of screening reliability.

Delayed protocol registration represents another limitation. Although registration occurred prior to data analysis and no post hoc modifications were made, delayed registration reduces transparency and introduces a theoretical risk of selective reporting.

Certainty of evidence, assessed using the GRADE approach, was low to very low across outcomes, reflecting the predominance of observational designs and variability in outcome reporting. Consequently, findings should be interpreted as hypothesis-generating rather than confirmatory.

Finally, practical requirements associated with propofol administration—particularly intravenous access, monitoring capability, and experienced personnel—may limit applicability in settings where subcutaneous administration is standard. These contextual factors should be considered when interpreting feasibility and generalizability.

Taken together, these strengths and limitations support cautious interpretation of findings within contemporary ethical and clinical frameworks emphasizing proportionality, shared decision-making, and symptom relief as core principles of palliative sedation.

### Implications for practice, policy, and future research

In clinical practice, propofol may be considered as a rescue therapy for palliative sedation in carefully selected refractory cases when standard sedatives prove inadequate. However, given the limited and heterogeneous evidence base, the feasibility and acceptability of its broader implementation remain uncertain. Its use requires careful titration, continuous monitoring for respiratory and hemodynamic effects, and administration by experienced multidisciplinary teams familiar with palliative sedation principles. Ethical safeguards, including explicit informed consent and clear documentation of decision-making processes, are essential to ensure proportional and ethically appropriate sedation.

The implementation of propofol in routine practice also requires specific infrastructure, including reliable intravenous access, continuous monitoring capability, and availability of appropriately trained personnel. These requirements may restrict its use to specialized palliative care units or hospital-based settings and reinforce its role primarily as a second- or third-line rescue option rather than a universally applicable first-line sedative.

From a policy perspective, clinical guidelines should incorporate clear recommendations regarding propofol use in palliative sedation, including indications for rescue therapy, dosing strategies, monitoring requirements, and safety protocols. Institutional policies should define required competencies and establish standardized documentation practices to support transparent and ethically robust decision-making. Appropriate training should include skills in intravenous infusion management, sedation titration, monitoring of physiological parameters, and ethical reasoning related to proportional sedation and consent.

Future research should prioritize well-designed multicenter studies to better characterize propofol use in diverse clinical settings and patient populations. In particular, prospective observational studies incorporating standardized reporting of dosing, monitoring, and ethical processes may represent feasible and ethically acceptable approaches to strengthening the evidence base. Further research should also explore patient, family, and caregiver perspectives to better define meaningful outcomes and indicators of high-quality palliative sedation.

Although standards for documentation and ethical decision-making in palliative sedation are already established, the variability observed across included studies suggests inconsistent reporting and implementation. This highlights the need for clearer operational guidance and standardized reporting frameworks specifically addressing propofol use in palliative sedation.

## Conclusions

This systematic review highlights propofol as a potential rescue option for palliative sedation in carefully selected refractory cases when conventional sedatives fail to provide adequate symptom relief. Propofol was associated with achievement of target sedation depth and reported symptom relief, either alone or in combination with other agents. However, substantial heterogeneity in study design, patient populations, dosing strategies, and outcome measurement limits the strength and comparability of these findings. Survival outcomes varied widely, and safety concerns—particularly respiratory depression—require careful clinical monitoring. Ethical challenges, including incomplete documentation of consent and occasional reports of life-shortening intention, further emphasize the importance of adherence to established safeguards such as proportional sedation, shared decision-making, and transparent documentation. Overall, the available evidence remains predominantly observational and of low to very low certainty, limiting firm conclusions regarding efficacy, safety, or survival outcomes. In practice, propofol should be considered primarily as a second- or third-line rescue therapy in settings with appropriate infrastructure, monitoring capability, and experienced multidisciplinary teams. Its broader implementation remains dependent on feasibility, institutional readiness, and resource availability.

Future research should prioritize prospective multicenter studies with standardized reporting of dosing, monitoring, clinical outcomes, and ethical processes, alongside evaluation of patient and family perspectives to better define meaningful indicators of high-quality palliative sedation.

Overall, propofol represents a potentially valuable rescue therapy for refractory suffering, but its use should remain guided by careful clinical judgment, structured governance, and continued efforts to strengthen the evidence base.

## Supplemental Material

sj-docx-1-pmj-10.1177_02692163261465774 – Supplemental material for Propofol for palliative sedation: A systematic review of clinical outcomes, safety, and ethical implications Supplemental material, sj-docx-1-pmj-10.1177_02692163261465774 for Propofol for palliative sedation: A systematic review of clinical outcomes, safety, and ethical implications by Luís Almeida-Jardim and Paulo Reis-Pina in Palliative Medicine

sj-docx-2-pmj-10.1177_02692163261465774 – Supplemental material for Propofol for palliative sedation: A systematic review of clinical outcomes, safety, and ethical implications Supplemental material, sj-docx-2-pmj-10.1177_02692163261465774 for Propofol for palliative sedation: A systematic review of clinical outcomes, safety, and ethical implications by Luís Almeida-Jardim and Paulo Reis-Pina in Palliative Medicine

## Data Availability

All data relevant to this study are included within the article. No additional datasets, code, or materials were generated or used.
