# Supplementary material for: Propofol for palliative sedation: A systematic review of clinical outcomes, safety, and ethical implications
Source: Palliat Med. 2026 Jul 31;40(9):1302–21. doi: 10.1177/02692163261465774 (PMC13612869; doi:10.1177/02692163261465774)
Supplement: Supplementary file 1 [file sj-docx-1-pmj-10.1177_02692163261465774.docx]

**Supplementary Table 1 - Database search strategies**

| **Databases** | **Search strings** | **Limits/Filters** |
| --- | --- | --- |
| MEDLINE (via PubMed) | ((((((("Palliative Care"[MeSH Terms] OR "Hospice Care"[MeSH Terms] OR "Palliative Care"[Title/Abstract] OR "Hospice Care"[Title/Abstract] OR "end-of-life care"[Title/Abstract] OR "terminal care"[Title/Abstract]) AND ("Propofol"[MeSH Terms] OR "Propofol"[Title/Abstract]) AND ("Palliative Sedation"[Title/Abstract] OR "terminal sedation"[Title/Abstract] OR "sedat*"[Title/Abstract])) NOT ("Intensive Care Units"[MeSH Terms] OR "ICU"[Title/Abstract] OR "intensive care"[Title/Abstract] OR "critical care"[Title/Abstract] OR "trauma"[Title/Abstract])) NOT ("review"[Publication Type] OR "systematic review"[Publication Type] OR "meta-analysis"[Publication Type])) AND ("Palliative Care"[MeSH Terms] OR "Hospice Care"[MeSH Terms] OR "Palliative Care"[Title/Abstract] OR "Hospice Care"[Title/Abstract] OR "end-of-life care"[Title/Abstract] OR "terminal care"[Title/Abstract]) AND ("Propofol"[MeSH Terms] OR "Propofol"[Title/Abstract]) AND ("Palliative Sedation"[Title/Abstract] OR "terminal sedation"[Title/Abstract] OR "sedat*"[Title/Abstract])) NOT ("Intensive Care Units"[MeSH Terms] OR "ICU"[Title/Abstract] OR "intensive care"[Title/Abstract] OR "critical care"[Title/Abstract] OR "trauma"[Title/Abstract])) NOT ("review"[Publication Type] OR "systematic review"[Publication Type] OR "meta-analysis"[Publication Type])) | Language: English; Publication date: 2005/01/01 to 2024/08/31 |
| Web of Science Core Collection | ("palliative care" OR "hospice care" OR "end-of-life care" OR "terminal care") (Topic) AND (sedat* OR "terminal sedation" OR "palliative sedation") (Topic) AND propofol (Topic) NOT (review OR "systematic review" OR "scoping review" OR "meta-analysis" OR book) (All Fields) NOT ("ICU" OR "intensive care" OR "critical care" OR "trauma care") (All Fields) | Language: English; Publication date: 2005/01/01 to 2024/08/31 |
| Scopus | ( TITLE-ABS-KEY ( ( "palliative care" OR "hospice care" OR "end-of-life care" OR "terminal care" ) ) AND TITLE-ABS-KEY ( propofol ) AND TITLE-ABS-KEY ( ( sedat* OR "terminal sedation" OR "palliative sedation" ) ) AND NOT TITLE-ABS-KEY ( ( "ICU" OR "intensive care" OR "critical care" OR "trauma care" ) ) AND NOT TITLE-ABS-KEY ( ( review OR "systematic review" OR "scoping review" OR "meta-analysis" OR book ) ) ) | Humans;  Language: English;  Document type: Article;  PUBYEAR: >2004 and <2025 |
