# Supplementary material for: Propofol for palliative sedation: A systematic review of clinical outcomes, safety, and ethical implications
Source: Palliat Med. 2026 Jul 31;40(9):1302–21. doi: 10.1177/02692163261465774 (PMC13612869; doi:10.1177/02692163261465774)
Supplement: Supplementary file 2 [file sj-docx-2-pmj-10.1177_02692163261465774.docx]

**Supplementary Table 2 – Examples of reported intravenous propofol dosing approaches for palliative sedation**

| **Study** | **Induction Bolus** | **Initial Infusion Rate** | **Maintenance Infusion** | **Titration Strategy** | **Notes** |
| --- | --- | --- | --- | --- | --- |
| Bodnar [9] | 0.5 mg/kg over 10 seconds (repeat every 5 min if needed; may escalate to 1.0 mg/kg) | Minimum 0.9 mg/kg/h (or 1.8 mg/kg/h if prior sedatives failed) | Continued infusion | Adjust every 20 min by 0.3 mg/kg/h; escalation up to 0.6–1.2 mg/kg/h increments | No maximum dose specified |
| Sulistio et al. [55] | 20 mg was given over 5 minutes | 1.0 mg/kg/h 2.0 | Dose range 60–90 mg/hour | Close nursing and physician monitoring were performed  until satisfactory sedation was achieved | Pain management was  maintained with continuous subcutaneous methadone infusion 10 mg/day |
| Herndon and Zimmerman [56] | Not reported | 10 mg/mL infused at 100 mg/h | Over the next 2 hours, the rate was increased to 390 mg/h | Titrating to comfort | The fentanyl drip (begun at 250 μg/h, reached 1000 μg/h) and lorazepam injections (4 mg/h) continued. |
| Torremorell and Casas [57] | 1.0–1.5 mg/kg | 2.0 mg/kg/h | Continued infusion | Increase by ~33% every 5 minutes until target sedation achieved | Protocol-based titration strategy |
| García Romo et al. [58] | Optional: 20 mg (50 kg) or 40 mg (75 kg) | 20–30 mg/h | Continued infusion | Increase by 20 mg every 5 minutes until refractory symptoms controlled | Weight-based fixed-dose approach |
| Cherny [59] | Not recommended | 0.3 mg/kg/h | 0.3–3.0 mg/kg/h | Increase by 0.3–0.6 mg/kg/h every 5–10 minutes | Widely cited proportional titration strategy |
| Krauss et al. [60] | Not specified | 0.3 mg/kg/h | Incremental increases from 0.3 mg/kg/h | Stepwise escalation according to sedation depth | Expert consensus recommending intravenous propofol use |
